# Supplementary material for: How wear, age, and sex relate to enamel chipping in Cayo Santiago rhesus macaques (Macaca mulatta)
Source: PLoS One. 2025 Dec 5;20(12):e0337554. doi: 10.1371/journal.pone.0337554 (PMC12680196; doi:10.1371/journal.pone.0337554)
Supplement: S1 Text — (DOCX) [file pone.0337554.s001.docx]

**Supplementary Information: Analysis by Tooth Type**

Here, we present results of a supplementary statistical analysis using separate logistic regression models for each tooth type and for wear vs. age separately. The purpose of this supplementary analysis was to eliminate any effect of collinearity between wear and age and to provide results by tooth type for readers interested in seeing how fixed effects varied in their impact across different tooth types.

**Methods**

Chipping was analyzed as a binary variable, with “0” representing no chips on a molar and “1” representing one or more chips on a molar. While performing multiple tests, as we do in this supplementary analysis, increases the chances of Type I error, there is longstanding and continuing controversy over when and how to use corrections for multiple tests. Some researchers, including statisticians, find the Bonferroni correction to be overly conservative and question whether corrections for multiple testing are necessary [1-3]. Armstrong [3] does not recommend its use except for situations in which it is “imperative to avoid Type I error” and when “…a large number of tests are carried out without preplanned hypotheses in an attempt to establish any results that may be significant” (2014:505). We believe that our study does not satisfy either of Armstrong’s [3] conditions for applying the Bonferroni correction or other corrections for reducing Type I error associated with multiple testing. Furthermore, given the low frequencies of chipping in our sample, the separate logistic regressions we conducted have low power such that to detect fixed effects (our goal), we did not consider it appropriate to correct for multiple testing. For these reasons, we use an alpha of 0.05.

To analyze the relationship between enamel chipping and tooth wear, we regressed enamel chipping on dental wear, sex, year-of-birth, and time spent in Sabana Seca for each of the six molar types (UM1, UM2, UM3, LM1, LM2, and LM3) using PROC LOGISTIC to conduct logistic regression in SAS 9.4. We refer to this model as “the full” model for wear. If year-of-birth and time spent in Sabana Seca were not significant predictors of chipping, we next ran a model for each of the six molar types with only wear, sex, and their interaction as predictors. Since we did not find interaction effects for wear and sex, we ran reduced models with only wear and sex as predictors. We report statistical results for the full models as well as the reduced models, identifying statistically significant predictors.

To analyze the relationship between enamel chipping and age, we regressed enamel chipping on age, sex, year-of-birth, and time spent in Sabana Seca for each of the six molar types (UM1, UM2, UM3, LM1, LM2, and LM3). We refer to this model for wear as “the full” model for age. As we did for wear, if year-of-birth and time spent in Sabana Seca were not significant predictors of chipping, we ran a reduced model for each of the six molar types with only age, sex, and their interaction as predictors. Again, because we did not find interaction effects, we ran the reduced model with only age and sex as predictors. We report the statistical results for the full model as well as the reduced model for age, identifying statistically significant predictors. We evaluated whether wear or age is a “better” predictor of enamel chipping by determining for how many of the six molar types we find a statistically significant effect for wear but not for age and vice versa. We evaluated the “sex” effect in all models (full and reduced model for wear; full and reduced model for age) to determine how often sex predicts chipping frequencies.

**Results**

Results for all regression models are provided in Supplementary Tables A-D, while a summary of the statistically significant results is given in Supplementary Table E. The full logistic regression models for wear were performed separately for each molar type with presence or absence of chipping regressed on wear (percentage of occlusal surface that was worn), year-of-birth, and time spent in Sabana Seca. Both wear and sex were statistically significant predictors for UM2 chipping, while sex and year of birth were statistically significant predictors for LM3 chipping.

Given that year of birth and time spent in Sabana Seca were not significant predictors for UM1, UM2, UM3, LM1, and LM2, reduced logistic regression models were run in which presence or absence of chipping was regressed only on wear and sex (interactions between them were not significant). In this set of five reduced models, wear emerged as a statistically significant predictor for both the UM1 and the UM2. In these reduced models, sex also emerged as a significant predictor for the UM2. The reduced model was not run for the LM3 because of the significant effect of year of birth in the full model. For wear, in all cases where a significant difference was found, wear increased the probability of chipping. For sex, in all cases of statistical significance, males had a higher probability of chipping than females. Finally, for year-of-birth, the probability of chipping decreased over time for the LM3.

The full logistic regression models for age were performed separately for each molar type with presence or absence of chipping regressed on age, year-of-birth, and time spent in Sabana Seca. In these models, only sex was statistically significant predictor for the LM3. Because year of birth and time spent in Sabana Seca were not significant predictors for any of the full regression models for age, reduced logistic regression models were run for all six molar types in which presence or absence of chipping was regressed only on age and sex (interactions between them were not significant). In this set of five reduced models, age emerged as a statistically significant predictor at the *p* < 0.05 level for the LM3.

**Table A: Full Logistic Regression Model Results for Percentage of Occlusal Surface that is Worn: Type 3 Analysis of Effects and Odds Ratios**

| Molar Type | Predictors | df | Wald Chi Square | p-value | Odds ratio point estimate | Odds ratio 95% confidence limits |
| --- | --- | --- | --- | --- | --- | --- |
| UM1 | Wear | 1 | 1.6531 | 0.1985 | 1.019 | 0.990-1.050 |
|  | Sex | 1 | 0.6916 | 0.4056 | 0.633 | 0.216-1.858 |
|  | Year-of-Birth | 1 | 0.9398 | 0.3323 | 0.946 | 0.846-1.058 |
|  | Time in Sebana Seca | 1 | 0.4136 | 0.5201 | 0.897 | 0.645-1.248 |
| UM2 | Wear | 1 | 6.6084 | 0.0138* | 1.059 | 1.012-1.109 |
|  | Sex | 1 | 4.0523 | 0.0441* | 0.106 (F<M) | 0.012-0.943 |
|  | Year-of-Birth | 1 | 0.0013 | 0.9710 | 1.003 | 0.854-1.178 |
|  | Time in Sebana Seca | 1 | 0.1036 | 0.7475 | 0.950 | 0.694-1.300 |
| UM3 | Wear | 1 | 0.1219 | 0.7270 | 1.012 | 0.947-1.081 |
|  | Sex | 1 | 0.9894 | 0.3199 | 0.417 | 0.074-2.339 |
|  | Year-of-Birth | 1 | 0.6748 | 0.4114 | 0.935 | 0.796-1.098 |
|  | Time in Sebana Seca | 1 | 2.1072 | 0.1466 | 1.142 | 0.955-1.081 |
| LM1 | Wear | 1 | 1.5645 | 0.2110 | 1.016 | 0.991-1.043 |
|  | Sex | 1 | 1.2561 | 0.2624 | 0.546 | 0.190-1.572 |
|  | Year-of-Birth | 1 | 0.0098 | 0.9213 | 0.995 | 0.893-1.107 |
|  | Time in Sebana Seca | 1 | 0.4547 | 0.5001 | 0.869 | 0.578-1.307 |
| LM2 | Wear | 1 | 1.3606 | 0.2434 | 1.022 | 0.985-1.059 |
|  | Sex | 1 | 0.0137 | 0.9067 | 1.076 | 0.314-3.692 |
|  | Year-of-Birth | 1 | 1.8749 | 0.1709 | 0.918 | 0.812-1.038 |
|  | Time in Sebana Seca | 1 | 0.0056 | 0.9405 | 0.991 | 0.775-1.267 |
| LM3 | Wear | 1 | 0.0873 | 0.7677 | 1.009 | 0.950-1.072 |
|  | Sex | 1 | 4.7341 | 0.0296* | 0.153 (F<M) | 0.028-0.830 |
|  | Year-of-Birth | 1 | 4.8384 | 0.0278* | 0.849 | 0.734-0.982 |
|  | Time in Sebana Seca | 1 | 3.6930 | 0.0546 | 1.264 | 0.995-1.605 |

* = significant at p < 0.05

**Table B: Reduced Model Results for Percentage of Occlusal Surface that is Worn: Type 3 Analysis of Effects and Odds Ratios**

| Molar Type | Predictors | df | Wald Chi Square | p-value | Odds ratio point estimate | Odds ratio 95% confidence limits |
| --- | --- | --- | --- | --- | --- | --- |
| UM1 | Wear | 1 | 4.3724 | 0.0365* | 1.028 | 1.002-1.055 |
|  | Sex | 1 | 0.5914 | 0.4419 | 0.671 | 0.242-1.856 |
| UM2 | Wear | 1 | 10.2770 | 0.0013* | 1.064 | 1.024-1.105 |
|  | Sex | 1 | 3.8996 | 0.0483* | 0.195 | 0.038-0.988 |
| UM3 | Wear | 1 | 1.0017 | 0.3169 | 1.028 | 0.974-1.085 |
|  | Sex | 1 | 0.2671 | 0.6053 | 0.686 | 0.164-2.864 |
| LM1 | Wear | 1 | 1.5688 | 0.2104 | 1.015 | 0.991–1.040 |
|  | Sex | 1 | 1.9102 | 0.1669 | 0.479 | 0.168- 1.361 |
| LM2 | Wear | 1 | 2.0282 | 0.1544 | 1.024 | 0.991-1.058 |
|  | Sex | 1 | 0.0082 | 0.9277 | 0.946 | 0.287-3.122 |
| LM3 | Wear | 1 | Reduced model was not run because of significant effect of year-of-birth in full model | | | |
|  | Sex | 1 |  | | | |

* = significant at p < 0.05**Table C: Full Logistic Regression Model Results for Age at Death: Type 3 Analysis of Effects and Odds Ratios**

| Molar Type | Predictors | df | Wald Chi Square | p-value | Odds ratio point estimate | Odds ratio 95% confidence limits |
| --- | --- | --- | --- | --- | --- | --- |
| UM1 | Age at Death | 1 | 0.5440 | 0.4608 | 1.044 | 0.931-1.170 |
|  | Sex | 1 | 0.9937 | 0.3188 | 0.571 | 0.190-1.718 |
|  | Year-of-Birth | 1 | 0.6969 | 0.4038 | 0.949 | 0.838-1.074 |
|  | Time in Sebana Seca | 1 | 0.6910 | 0.4058 | 0.861 | 0.605-1.225 |
| UM2 | Age at Death | 1 | 0.7006 | 0.4026 | 1.063 | 0.921-1.227 |
|  | Sex | 1 | 4.8759 | 0.0272 | 0.089 | 0.010-0.762 |
|  | Year-of-Birth | 1 | 0.6646 | 0.4150 | 0.933 | 0.791-1.101 |
|  | Time in Sebana Seca | 1 | 0.0213 | 0.8838 | 1.020 | 0.784-1.327 |
| UM3 | Age at Death | 1 | 1.3854 | 0.2392 | 1.105 | 0.936-1.304 |
|  | Sex | 1 | 1.3987 | 0.2369 | 0.347 | 0.060-2.006 |
|  | Year-of-Birth | 1 | 0.0177 | 0.8941 | 0.988 | 0.832-1.175 |
|  | Time in Sebana Seca | 1 | 1.2493 | 0.2637 | 1.111 | 0.924-1.336 |
| LM1 | Age at Death | 1 | 0.3080 | 0.5789 | 1.030 | 0.928-1.144 |
|  | Sex | 1 | 1.2542 | 0.2627 | 0.533 | 0.177-1.603 |
|  | Year-of-Birth | 1 | 0.0013 | 0.9709 | 0.998 | 0.886-1.124 |
|  | Time in Sebana Seca | 1 | 0.5502 | 0.4582 | 0.842 | 0.535-1.326 |
| LM2 | Age at Death | 1 | 1.1765 | 0.2781 | 1.083 | 0.938-1.250 |
|  | Sex | 1 | 0.0496 | 0.8237 | 0.867 | 0.247-3.046 |
|  | Year-of-Birth | 1 | 0.5985 | 0.4392 | 0.946 | 0.823-1.088 |
|  | Time in Sebana Seca | 1 | 0.0335 | 0.8549 | 0.977 | 0.761-1.254 |
| LM3 | Age at Death | 1 | 1.1628 | 0.2809 | 1.080 | 0.939-1.241 |
|  | Sex | 1 | 6.1364 | 0.0132* (F<M) | 0.109 | 0.019-0.630 |
|  | Year-of-Birth | 1 | 2.7320 | 0.0984 | 0.877 | 0.750-1.025 |
|  | Time in Sebana Seca | 1 | 3.5240 | 0.0605 | 1.244 | 0.990-1.563 |

* = significant at p < 0.05

**Table D: Reduced Model Results for Age-at-Death two predictors**

| Molar Type | Predictors | df | Wald Chi Square | p-value | Odds ratio point estimate | Odds ratio 95% confidence limits |
| --- | --- | --- | --- | --- | --- | --- |
| UM1 | Age at Death | 1 | 2.1195 | 0.1454 | 1.069 | 0.977-1.170 |
|  | Sex | 1 | 0.9610 | 0.3269 | 0.597 | 0.212-1.676 |
| UM2 | Age at Death | 1 | 3.2578 | 0.0711 | 1.107 | 0.991-1.236 |
|  | Sex | 1 | 4.7608 | 0.0291* (F<M) | 0.168 | 0.034-0.834 |
| UM3 | Age at Death | 1 | 3.1947 | 0.4329 | 1.128 | 0.988-1.287 |
|  | Sex | 1 | 0.6149 | 0.0739 | 0.554 | 0.126-2.427 |
| LM1 | Age at Death | 1 | 0.3349 | 0.5628 | 1.026 | 0.941-1.118 |
|  | Sex | 1 | 1.7003 | 0.1922 | 0.488 | 0.166-1.434 |
| LM2 | Age at Death | 1 | 2.9965 | 0.0834 | 1.113 | 0.986-1.255 |
|  | Sex | 1 | 0.1749 | 0.6758 | 0.769 | 0.224-2.639 |
| LM3 | Age at Death | 1 | 6.3739 | 0.0116* | 1.153 | 1.032-1.288 |
|  | Sex | 1 | 6.2172 | 0.0127* (F<M) | 0.157 | 0.037-0.673 |

* = significant at p < 0.05

**Table E: Summary of statistically significant results from all logistic regression models (full results shown in Supplemental Tables (S1-S4)**

| Full logistic model: Presence of chips as a function of wear, sex, year-of-birth, time in Sebana Seca | Significant at p < 0.05 |
| --- | --- |
| UM1 | - |
| UM2 | Wear (p=0.0138), Sex (p=0.0441) |
| UM3 | - |
| LM1 | - |
| LM2 | - |
| LM3 | Sex (p=0.0296), YOB (p=0.0278) |
| Reduced logistic model - Presence of chips as a function of wear and sex | Significant at p < 0.05 |
| UM1 | Wear (p=0.0365) |
| UM2 | Wear (p=0.0013), Sex (p=0.0483) |
| UM3 | - |
| LM1 | - |
| LM2 | - |
| LM3 | Not run because of significant effect of YOB |
| Full logistic model: Presence of chips as a function of age-at-death, sex, year-of-birth, time in Sebana Seca | Significant at p < 0.05 |
| UM1 | - |
| UM2 | - |
| UM3 | - |
| LM1 | - |
| LM2 | - |
| LM3 | Sex (p=0.0132)* |
| Reduced logistic model - Presence of chips as a function of age-at-death and sex | Significant at p < 0.05* |
| UM1 | - |
| UM2 | - |
| UM3 | - |
| LM1 | - |
| LM2 | - |
| LM3 | Sex (p=0.0127)*, Age-at-Death (p=0.0116)* |

**Summary and Discussion**

Overall, the supplementary analysis by tooth type we report in the SI supports the GLMM analysis we reported in the main text. For both this supplementary analysis and the GLMM analysis, wear and sex emerge as the most common predictors of chipping frequencies (Table S5). Wear is a better predictor than age: it is significant for two tooth types (UM1, UM3) while age-at-death is significant for one only tooth type (LM3). Moreover, we note that for the LM3 we did not perform a reduced model of the regression of chipping on sex and wear owing to the significant effect of year-of-birth. For this reason, we do not know if wear, in addition to age-at-death, might also have been a significant predictor of LM3 chipping in the reduced model.

The analysis by tooth type additionally reveals a possible effect in the LM3 for year-of-birth (in the full regression model for wear). It is not clear why enamel chipping would have decreased over time for the LM3 in Cayo Santiago rhesus macaques. It is possible this finding is related to subtle changes in diet and geophagic behavior of these monkeys over the time span encompassed by this study, but we have no direct evidence of these changes.

Lastly, it is notable that significant relationships between chipping and predictors vary by tooth type. Reasons for the variability of these relationships across tooth types may have to do with differences in eruption ages, with third molars erupting later in development, as well as enamel thickness, with third molars having thicker enamel than first and second molars, at least in humans [4]. Both of these factors would tend to decrease the frequency of chipping on third molars and could affect relationships between chipping and other model predictors. Although the GLMM reported in the main text did not reveal an effect of tooth type on chipping, the three lowest values for chipping frequencies in our sample all occur on third molars, suggesting a possible effect of molar type on chipping frequencies and the relationship between chipping and other predictors, such as dental wear or age.

**References**

1. Cabin RJ, Mitchell RJ. To Bonferroni or not to Bonferroni: When and how are the questions. Bull Ecol Soc Amer. 2000;81(3):246-8.
2. Streiner DL, Norman GR. Correction for multiple testing: Is there a resolution?. Chest. 2011; 140(1):16-8.
3. Armstrong RA. When to use the Bonferroni correction. Ophthalmic Physiol Opt 2014; 34(5):502-8.
4. Smith TM, Olejniczak AJ, Reid DJ, Ferrell RJ, Hublin JJ. Modern human molar enamel thickness and enamel–dentine junction shape. Arch Oral Biol. 2006;51(11):974-95.
