# Supplementary material for: How wear, age, and sex relate to enamel chipping in Cayo Santiago rhesus macaques (Macaca mulatta)
Source: PLoS One. 2025 Dec 5;20(12):e0337554. doi: 10.1371/journal.pone.0337554 (PMC12680196; doi:10.1371/journal.pone.0337554)
Supplement: S2 Text — (DOCX) [file pone.0337554.s002.docx]

**Metadata**

**Maxillary molars**

| Column | Heading | Description |
| --- | --- | --- |
| A | Museum.number | Museum number given to specimen |
| B | CsUniId | Unique identifier of Cayo Santiago specimen |
| C | YOB | Year of birth |
| D | Sex | Female (F) or Male (M) |
| E | Age.at.Death.yrs | Age at death in years |
| F | SS Trans | Age at which individual was transferred from Cayo Santiago to Sabana Seca |
| G | Time in SS | Length of time individual spent in Sebana Seca |
| H | Side.Quantified.For.Wear | Side of dentition on which wear was quantified |
| I | UM1PerWear | Area of dentine exposure as a percentage of occlusal area for upper first molar (UM1) |
| J | UM2PerWear | Area of dentine exposure as a percentage of occlusal area for upper second molar (UM2) |
| K | UM3PerWear | Area of dentine exposure as a percentage of occlusal area for upper third molar (UM3) |
| L | Chips_UM1 | Presence or absence of chips on upper first molar (UM1) |
| M | UM1h(largest if more than 1) | Value of “h” (see text) for the largest chip on the upper first molar (UM1) in mm. |
| N | UM1CA (largest if more than 1) | Area of the largest chip on the upper first molar (UM1) in mm^2^ |
| O | UM1OA | Occlusal Area of the upper first molar (UM1) in mm^2^ |
| P | UM1hOA | 100* the value of “h” for the upper first molar (UM1) divided by its occlusal area |
| Q | UM1CAOA | 100* the area of the largest chip on the upper first molar (UM1) divided by its occlusal area |
| R | Chips_UM2 | Presence or absence of chips on upper second molar (UM2) |
| S | UM2h(largest if more than 1) | Value of “h” (see text) for the largest chip on the upper second molar (UM2) in mm. |
| T | UM2CA (largest if more than 1) | Area of the largest chip on the upper second molar (UM2) in mm^2^ |
| U | UM2OA | Occlusal Area of the upper second molar (UM2) in mm^2^ |
| V | UM2hOA | 100* the value of “h” for the upper second molar (UM2) divided by its occlusal area |
| W | UM2CAOA | 100* the area of the largest chip on the upper second molar (UM2) divided by its occlusal area |
| X | Chips_UM3 | Presence or absence of chips on upper third molar (UM3) |
| Y | UM3h(largest if more than 1) | Value of “h” (see text) for the largest chip on the upper third molar (UM3) in mm. |
| Z | UM3CA (largest if more than 1) | Area of the largest chip on the upper third molar (UM3) in mm^2^ |
| AB | UM3OA | Occlusal Area of the upper third molar (UM3) in mm^2^ |
| AC | UM3hOA | 100* the value of “h” for the upper third molar (UM3) divided by its occlusal area |
| AD | UM3CAOA | 100* the area of the largest chip on the upper third molar (UM3) divided by its occlusal area |

**Mandibular molars**

| Column | Heading | Description |
| --- | --- | --- |
| A | Museum.number | Museum number given to specimen |
| B | CsUniId | Unique identifier of Cayo Santiago specimen |
| C | YOB | Year of birth |
| D | Sex | Female (F) or Male (M) |
| E | Age.at.Death.yrs | Age at death in years |
| F | SS Trans | Age at which individual was transferred from Cayo Santiago to Sabana Seca |
| G | Time in SS | Length of time individual spent in Sebana Seca |
| H | Side.Quantified.For.Wear | Side of dentition on which wear was quantified |
| I | LM1PerWear | Area of dentine exposure as a percentage of occlusal area for lower first molar (LM1) |
| J | LM2PerWear | Area of dentine exposure as a percentage of occlusal area for lower second molar (LM2) |
| K | LM3PerWear | Area of dentine exposure as a percentage of occlusal area for lower third molar (LM3) |
| L | Chips_LM1 | Presence or absence of chips on lower first molar (LM1) |
| M | LM1h(largest if more than 1) | Value of “h” (see text) for the largest chip on the lower first molar (LM1) in mm. |
| N | LM1CA (largest if more than 1) | Area of the largest chip on the lower first molar (LM1) in mm^2^ |
| O | LM1OA | Occlusal Area of the lower first molar (LM1) in mm^2^ |
| P | LM1hOA | 100* the value of “h” for the lower first molar (LM1) divided by its occlusal area |
| Q | LM1CAOA | 100* the area of the largest chip on the lower first molar (LM1) divided by its occlusal area |
| R | Chips_LM2 | Presence or absence of chips on lower second molar (LM2) |
| S | LM2h(largest if more than 1) | Value of “h” (see text) for the largest chip on the lower second molar (LM2) in mm. |
| T | LM2CA (largest if more than 1) | Area of the largest chip on the lower second molar (LM2) in mm^2^ |
| U | LM2OA | Occlusal Area of the lower second molar (LM2) in mm^2^ |
| V | LM2hOA | 100* the value of “h” for the lower second molar (LM2) divided by its occlusal area |
| W | LM2CAOA | 100* the area of the largest chip on the lower second molar (UM2) divided by its occlusal area |
| X | Chips_LM3 | Presence or absence of chips on lower third molar (LM3) |
| Y | LM3h(largest if more than 1) | Value of “h” (see text) for the largest chip on the lower third molar (LM3) in mm. |
| Z | LM3CA (largest if more than 1) | Area of the largest chip on the lower third molar (LM3) in mm^2^ |
| AB | LM3OA | Occlusal Area of the lower third molar (LM3) in mm^2^ |
| AC | LM3hOA | 100* the value of “h” for the lower third molar (LM3) divided by its occlusal area |
| AD | LM3CAOA | 100* the area of the largest chip on the lower third molar (UM3) divided by its occlusal area |
