# Supplementary material for: How wear, age, and sex relate to enamel chipping in Cayo Santiago rhesus macaques (Macaca mulatta)
Source: PLoS One. 2025 Dec 5;20(12):e0337554. doi: 10.1371/journal.pone.0337554 (PMC12680196; doi:10.1371/journal.pone.0337554)
Supplement: Attachment — Submitted filename: Letter to Editor.docx [file pone.0337554.s006.docx]

**Response to Editor**

Thank you for your careful review of our paper. Reviewers had minor revisions, which we have addressed in the revised MS. Below, we detail the changes that were made in this version of our MS.

REVIEWER 1: The authors have done an excellent job of addressing my concerns. I have only a small number of minor notes on this version of the manuscript:
Page 12, line 257: “chip occlusal areas of” seems to have an unnecessary “of”
Page 14, line 294: “are” should be “as” in this line

RESPONSE: These two items have been corrected

REVIEWER 1: Page 18: p-values throughout are listed as “p < number” when they may be better listed as “p = number”

RESPONSE: We originally reported p-values with “=” signs as they are exact p-values. Reviewer 2 suggested we change them to inequalities. Reviewer 1 (as noted above), however, states that these should be listed as “p=number” (which is what we thought originally). We have looked further into this issue, and it seems Reviewer 1 is correct: these should be reported as exact p-values with an equal sign. We have made this change throughout the MS.

RESPONSE: These two items have been corrected

REVIEWER 1: Page 25, lines 471-473: This sentence seems to have a misplaced comma

RESPONSE: Corrected.

REVIEWER #2: This paper is well written, and the authors have incorporated the reviewers’ comments thoroughly. The revised analyses and results are explained in a clear manner. I appreciate that the authors included the original results in the supplemental material for those interested in the analysis of individual teeth. My original comments were well addressed. I have only two minor comments, both related to figure legends.
Figure 1 legend: the c figure is included twice. Should these descriptions be combined? Also, the colors used in the figure are quite similar. Perhaps either colors that contrasting more, or include one of the colors by dotted lines for individuals with color blindness.

RESPONSE: We are showing something different in “c” so we prefer to leave “c” as it is. We agree that the green and blue in this figure would be problematic for individuals with blue-yellow color blindness. We have changed the colors in Figure 1 to blue/red.

REVIEWER #2: Figure 2 legend – The first part of this figure legend is awkward. It seems that there is either a word missing, or extra words.

RESPONSE: Sorry, we are not seeing where the reviewer finds the wording to be awkward. For this reason, we have not changed this figure legend.

Many thanks for your time and for helping us improve this MS.

Best wishes,

Debbie Guatelli-Steinberg and co-authors
